# The exposure of field-grown maize seedlings to weed volatiles affects their growth and seed quality

**DOI:** 10.3389/fpls.2023.1141338

**Published:** 2023-08-15

**Authors:** Yusuke Sakurai, Satomi Ishizaki, Shota Izumi, Takuma Yoshida, Kaori Shiojiri, Junji Takabayashi

**Affiliations:** ^1^ Graduate School of Science and Technology, Niigata University, Niigata, Japan; ^2^ Department of Agriculture, Ryukoku University, Otsu, Japan; ^3^ Center for Ecological Research, Kyoto University, Otsu, Japan

**Keywords:** plant-plant communication, mugwort, goldenrod, maize, salicylic acid, sugar content, weed volatiles, seed quality

## Abstract

Plants exposed to volatiles emitted from artificially damaged conspecific or heterospecific plants exhibit increased resistance to herbivorous insects. Here, we examined whether volatiles from artificially damaged weeds affect maize growth and reproduction. Seven days after germination, maize seedlings were exposed to volatiles emitted by artificially damaged mugwort (*Artemisia indica* var. *maximowiczii*) or tall goldenrod (*Solidago altissima*) plants either separately, or as a mixture of the two, for seven days. Unexposed seedlings were used as controls. Treated and control seedlings were cultivated in an experimental field without any insecticides applied. Plants exposed to either of the three volatile treatments sustained significantly less damage than controls. Additionally, seedlings exposed to either goldenrod or mixed volatiles produced more leaves and tillers than control plants. Furthermore, a significant increase in the number of ears was observed in plants exposed to the volatile mixture. In all treated plants, ear sugar content was significantly higher than that in the controls. Further, we cultivated seedlings that were either exposed to the volatile mixture or unexposed, under the conventional farming method using pesticides. Similar significant differences were observed for sugar content, number of tillers, leaves, damaged leaves, and ears. Laboratory experiments were conducted to further evaluate the mechanisms involved in the improved performance of volatile-treated plants. A significant reduction in the growth of common armyworm (*Mythimna separata*) larvae was observed when maize plants were exposed to the volatile mixture. This treatment did not affect the amount of jasmonic acid in the seedlings, whereas salicylic acid content increased upon exposure. The characteristic differences in chemical composition of mugwort and goldenrod volatiles were confirmed and, in turn, the volatile mixture differed significantly from the volatiles of either species.

## 1 Introduction

In response to the damage caused by herbivorous arthropods, plants emit a bouquet of volatiles (herbivory-induced plant volatiles (HIPVs)) that attract carnivorous natural enemies of these herbivores (for review, Dicke et al., 1990; Turlings and Erb, 2018; Takabayashi and Shiojiri, 2019; Takabayashi, 2022). Further, when uninfested conspecific or heterospecific plants are exposed to HIPVs, they become more resistant to herbivores, a phenomenon known as “plant-plant communication” which is mediated by HIPVs (for review Karban et al., 2014; Yoneya and Takabayashi, 2014; Meents and Mithöfer, 2020). Furthermore, plant-plant communication can be also mediated by volatiles emitted from artificially damaged plants (Dolch and Tscharntke, 2000; Karban et al., 2006; Kikuta et al., 2011; Hagiwara et al., 2021). Green leaf volatiles (GLVs) composed of C6 aldehyde, alcohol, and acetate, are involved in both HIPVs and artificially damaged-plant volatiles, and some GLVs individually mediate plant-plant communication in several plant species, such as lima bean (Arimura et al., 2001), *Arabidopsis thaliana* (e.g. Kishimoto et al., 2005; Kishimoto et al., 2006), and maize (e.g. Engelberth et al., 2004; Ruther and Kleier, 2005). Plants also respond to volatile terpenoids (Maffei, 2012). Arimura et al. (2000) reported that (*E*)-*β*-ocimene, (*E*)-4,8-dimethyl-1,3,7-nonatriene, and (*E,E*)-4,8,12-trimethyl-1,3,7,11-tridecatetraene emitted from lima bean leaves infested by two-spotted spider mites (*Tetranychus urticae*) induced defensive genes in uninfested lima bean leaves. Terpenes such as 1,8-cineole and *β* -caryophyllene, which are included in volatiles released by artificially damaged sagebrush, induce defense against leaf herbivore in neighboring intact conspecifics (Shiojiri et al., 2015).

Plant-plant communication is a recently discovered defensive strategy in plants, which presumably might be used in ecofriendly crop production. Shiojiri et al. (2017); Shiojiri et al. (2020b) reported that artificial exposure of soybean plants to volatiles from mechanically damaged goldenrods for three weeks early in development resulted in the reduction of damaged leaves and seeds and the increase in seed isoflavone and saponin contents. Additionally, they reported that a three-week exposure of rice seedlings to volatiles from artificially damaged weeds in a nursery resulted in the reduction of damaged leaves and an increase in the number of grains per plant (Shiojiri et al., 2020a).

In this study, we focused on maize, one of the most cultivated crops worldwide. Previous laboratory studies have shown that plant-plant communication in maize is mediated by plant volatiles (Engelberth et al., 2004; Ruther and Kleier, 2005). Here, we conducted field and laboratory experiments to evaluate the effects of artificially damaged-weed volatiles on maize production. We used two weed species (mugwort and goldenrod) and examined whether volatiles from artificially damaged weeds, independently or as a mixture, affected defensive responses, growth, ear production, and seed quality in maize plants.

## 2 Materials and methods

### 2.1 Plants

**Figure 1:**
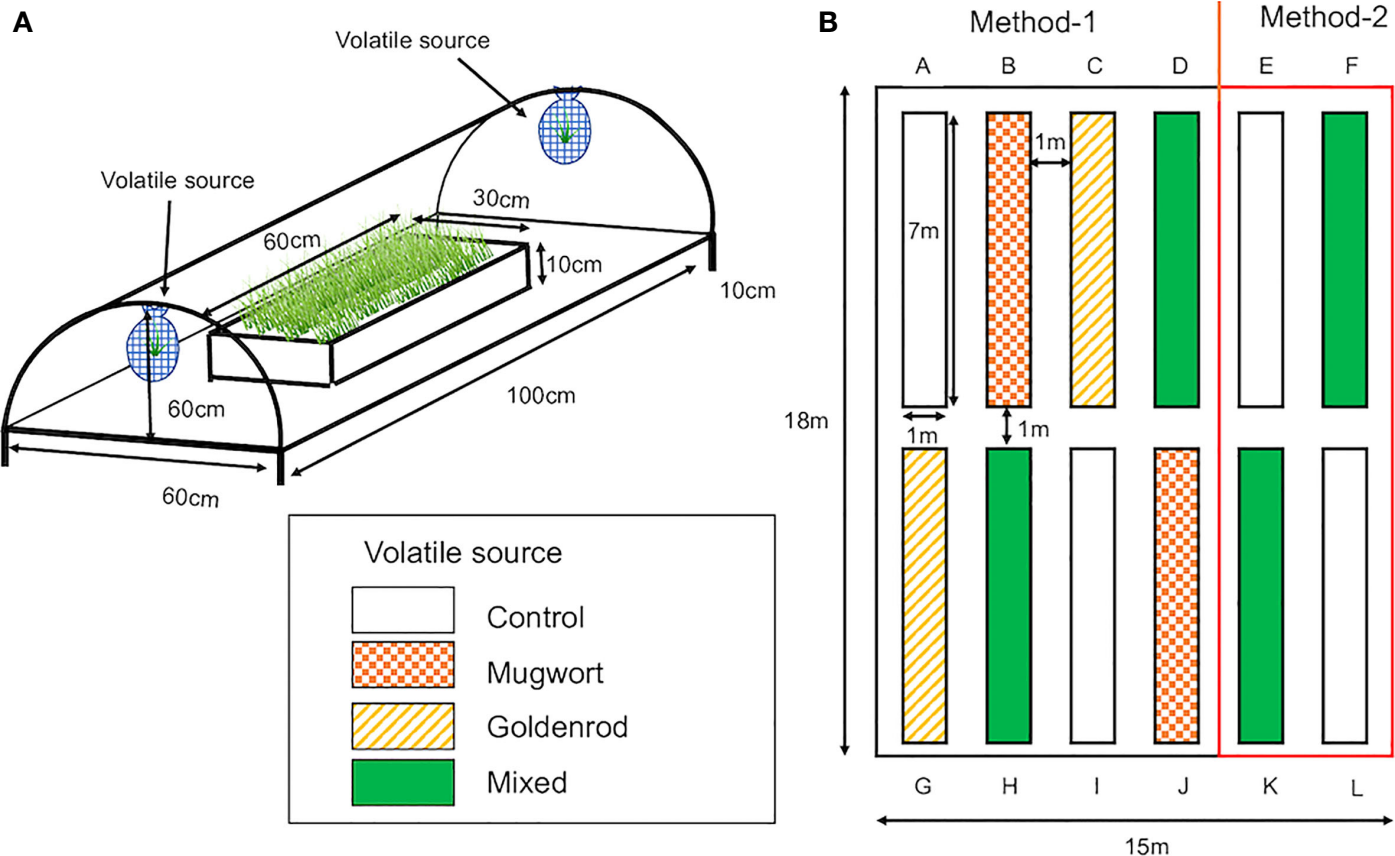
**(A)** Plastic tunnels used for the exposure of maize seedlings to weed volatiles under both laboratory and field conditions. Tunnels were made of garden poles and covered with clear plastic sheets. Two mesh nets, in which cut weed plants were placed, were hung on both sides of the tunnel. The tunnel was kept at a height of 10 cm above the ground for ventilation. **(B)** Layout of rows in which volatile-exposed and control seedlings were planted individually in the field. Control seedlings were planted in rows (A, E, I, L). Seedlings exposed to mugwort volatiles were planted in rows (B, J) Seedlings exposed to goldenrod volatiles were planted in rows (C, G), and seedlings exposed to the mixed volatiles were planted in rows (D, F, H, K) Seedlings in rows (A-D, G-J) were cultivated using Method-1 and those in the rest of the rows by Method-2.

Maize (*Zea mays* var. *saccharata*, Canberra 90EX) seeds were obtained from Takii Seed Corporation (Kyoto, Japan) and sown in 128-hole trays (30 × 60 cm; hole size: 3 cm diameter and 4.5 cm depth; Home Center Musashi, Niigata, Japan) filled with potting soil to a depth of 3.5 cm and covered with 1 cm of porous silicate (Inenica Krion Corporation, Aichi, Japan). The trays were kept in the laboratory under a 16/8 h photoperiod at about 25°C for two days to confirm germination. Then, the trays were placed in a plastic tunnel (100 cm long, 60 cm wide, and 60 cm high; **Figure 1A**) installed in the field at the experimental station of Niigata University in Niigata City, Japan, in June 2022 for field experiments, and in a climate-controlled chamber (Growth chamber, SANYO, Osaka, Japan) under a 16/8 h photoperiod at 25 ± 3°C, for laboratory experiments.

### 2.2 Insects

Common armyworm (*Mythimna separata*, Lepidoptera: Noctuidae) eggs were transferred from the Center of Ecological Research, Kyoto University, to the laboratory at Niigata University in 2022. After hatching, larvae were reared on an artificial diet (Insecta LF, Nosan Corporation, Kanagawa, Japan) in lidded plastic cups in a climate chamber set at 25 ± 3°C, under a 16/8 h photoperiod and 50%-70% relative humidity. Second stadium larvae were used for the experiments.

### 2.3 Volatile sources

Mugwort (*Artemisia indica* var. *maximowiczii*) and tall goldenrod (*Solidago altissima*), were collected from the Niigata University campus. The former is native to Japan, while the latter is an invasive species originated in North America. Both species were found on campus in open spaces such as along roadsides and ridges, more than 100 m away from the experimental fields. To prepare the volatile sources, five plants of each of the two selected weed species were cut finely with scissors. Cut weeds (ca. 30 g) were placed in a fine plastic mesh net (110 mm × 210 mm, mesh size 0.5 mm, Komeri Corporation, Niigata, Japan) as volatile sources. Experimental treatments included mugwort volatiles, goldenrod volatiles, and volatile mixture.

### 2.4 Exposure to weed volatiles

For field experiments, on the 7th day of germination, 120 maize seedlings were exposed for seven days to volatiles emitted from ca. 30 g of mugwort (mugwort-volatiles), or those emitted from ca. 30 g of goldenrod (goldenrod-volatiles), or to a mixture of the two weeds (ca. 15 g each) (volatile mixture). Control seedlings were not exposed to any volatile source during these seven days. For the laboratory experiments, we selected mixed weeds for volatile source due to the lack of experimental space for other odor sources.

Seedling exposure to the volatile sources was conducted in a plastic tunnel (100 cm long, 60 cm wide, 60 cm high) on a cultivation tray either in the field for field experiments or in a climate-controlled room for laboratory experiments under a light intensity of 900 lux, a 16/8 h photoperiod, and 25 ± 3°C temperature (**Figure 1A**). Trays were placed 150 cm apart with a polypropylene shielding plate (0.9 m × 1.8 m × 3 mm thickness) set between adjacent trays. For exposure, two mesh nets as volatile sources were hung in the tunnel, approximately 10 cm above the seedlings, for seven days. Volatile sources were replaced by new ones at 2-day intervals. Empty nets were hung above control seedlings. For laboratory experiment, after 7-day exposure, seedlings were individually transplanted to plastic pots (9.0 cm in diameter 7.5 cm in height) filled with potting soil (Takii Seed Corporation), vermiculite (Komeri Corporation, Niigata, Japan), arbuscular mycorrhiza (Matsumoto Institute of Microorganisms Co., Ltd), and added chemical fertilizer (Takamura Corporation, Tochigi, Japan) in a volume ratio of 1:1:0.01:0.01, and grown in a climate-controlled chamber at a temperature of 25 ± 3.5°C, under a 16/8 h photoperiod.

### 2.5 Field experiments

Twelve rows (7 × 1 m each) were made in the experimental field (18 × 15 m) at Niigata University (**Figure 1B**). The rows were 1 m apart and covered with black plastic film (0.02 mm × 1250 mm × 200 m, Komeri Corporation, Niigata, Japan) with 14 × 2 holes at 50 cm intervals for planting. All the rows were surrounded and covered by a net (1.5 m high × 50 m long; Komeri Corporation, Niigata, Japan).

Seedlings were exposed to the volatile sources in mid-May 2022. Then, after 7 d of exposure, they were planted in rows. The arrangement of treated and control seedlings is shown in **Figure 1B**. After 24 and 48 days from planting, approximately 60 g of fertilizer (N:P:K, 14:10:13, Takamura Corporation, Tochigi, Japan) was added to each row. Plants were watered daily using an automated sprinkle-irrigation system.

Maize plants were cultivated in the experimental field using two methods; Method-1: without pesticides and ear picking, and Method-2: using pesticides and by picking multiple ears based on standard maize cultivation methods, i.e., two rows, one for control and the other for volatile mixture-treated seedlings. In Method-2, 1 liter of BT-agent (1000× dilution, Sumitomo Chemical Co., Ltd., Tokyo, Japan) was sprayed after 46 days from planting, and both BT agent and Cartap granule (Padang granule, 40 g per row, Sumitomo Chemical Co., Ltd., Tokyo, Japan) were used after 52 days. Except for the top ear, multiple ears were counted and picked daily throughout the maturation period.

The number of tillers, intact, and damaged leaves were recorded on the 19th day and the number of ears formed on each plant was recorded after 62 days from planting. Mature top ears were harvested after 71 days, in late July 2022. For maize cultivated using Method-1, we harvested the second and third ears 10 days after harvesting the top ears. Ear length, total number of kernels, sugar content, and kernel dry weight were measured. The number of larvae of Asian corn borer (*Ostrinia furnacalis*) in the top ears was observed under the naked eye. Larvae inside the ears were picked with a long bamboo needle for counting. All ear measurements were performed within two days after harvest.

To calculate the number of kernels per ear, we arbitrarily selected three rows on each ear to count the number of kernels per row. The mean number of kernels per row was multiplied by the number of rows per ear. The percentage of mature seeds in the second and third ears of the plants cultivated using Method-1 was also determined. Seed maturity was scored based on the intensity of their yellow color from 0 to 1 (0.5 notch) under the naked eye.

### 2.6 Kernel sugar content and taste assessment

One of the ears located at the top of the maize plant was divided into three parts, and 15 kernels were taken from the middle part. Five kernels were crushed, and the juice of 0.5 ml was subjected to sugar content measurement using a sugar meter (PORTABLE BRIX METER, Kyoto Electronics Industry Corporation, Kyoto, Japan). The rest of the kernels were oven-dried at 50°C (KES-501CT, KITAZAWA SANGYO Co., LTD, Tokyo, Japan) for one week and weighed using a fine balance.

For the evaluation of taste, kernels from volatile-exposed and control plants cultivated using Method-2 were boiled for 5 min. Boiled kernels were offered to 21 persons to taste their sweetness using a blind test method.

### 2.7 Feeding experiments using *M. separata* larvae under laboratory conditions

Twenty-four seedlings exposed to the volatile mixture and control seedlings were used in a feeding experiment. One, second-instar larva weighing approximately 2 mg was allowed to feed on either a volatile-exposed or a control seedling covered with a cylindrical clear plastic sheet (10 cm in diameter) with a piece of fine plastic net at the top to prevent possible volatile-mediated communication between plants. Each seedling infested with a larva was then incubated in a climate-controlled room at a temperature of 25°C ± 3.5°C under a 16/8 h photoperiod. Larvae were weighed daily for five days.

To measure seedling growth, we sampled exposed and control seedlings on the 7th and 14th days after infestation with the larvae. Seedlings were dried in an incubator at 50°C for 4 days and weighed using an electronic balance with 0.1 mg precision.

### 2.8 Quantification of jasmonic acid (JA) and salicylic acid (SA)

One leaf from a maize plant exposed to the volatile mixture for seven days and a control plant was collected to determine JA and SA content using a liquid chromatography-tandem mass spectrometry (LC/MS/MS) system after Ozawa et al. (2000). We repeated the analyses 12 times.

### 2.9 Volatile analysis

We placed either goldenrod (3 g), cut mugwort (3 g), or a mixture (1.5 g of each) of the two in a 2 L separable flask (ca.12.5 cm diam., 23 cm height) immediately after cutting. Purified air (100 mL/min airflow) was passed through the flask, and the headspace volatiles were collected on porous polymer beads (Tenax-TA, 100 mg, mesh 20/35, GL Sciences Inc., Tokyo, Japan) in a glass tube (3.0 mm internal diameter, 160 mm length) for 1 h (N = 4). The collected volatiles were analyzed using a gas chromatograph-mass spectrometer (GCMS) equipped with an HP-5MS capillary column (Agilent Technologies Inc., Santa Clara, CA, USA, HP-5MS 30m) and a thermo-desorption cold-trap injector (GL Science TD2530). The temperature of the oven in the gas chromatograph was programmed to increase from 40°C (5-min hold) to 280°C at a rate of 15°C/min. Tentative compound identification was performed by comparison of experimental mass spectra against a commercial spectral library (Wiley7N, John Wiley & Sons Inc., Hoboken, NJ, United States). Total ion intensities were used to compare the amounts of each compound between treatments.

### 2.10 Statistical analysis

All statistical analyses were conducted using R ver. 4.0.3 (R Core Team, 2020). First, treatment effects were analyzed by the Tukey-HSD test. To meet the assumptions of normally distributed residuals, the total number of tillers, leaves, and ears, and ear length and number of larvae of Asian corn borer in the top ear were log-transformed, and the rate of damaged leaves per plant was arcsine square-root transformed prior to analysis. Larval weights were analyzed using the *t*-test for each day with the Bonferroni correction. As for the results of the taste assays, these were analyzed using a binomial test. With respect to the composition of volatiles, we calculated Bray–Curtis dissimilarity indices based on the relative amount of each volatile detected in the GC–MS analysis to compare the differences in composition between mugworts and goldenrods, and their mixture. A permutational multivariate analysis of variance (PERMANOVA) of the Bray–Curtis index was further performed to test for significant differences in scent composition (10 000 permutations). To visualize the distribution of scent profiles in the volatile space, we conducted nonmetric multidimensional scaling (NMDS) analysis based on the Bray–Curtis dissimilarity indices. Bray–Curtis/NMDS and PERMANOVA were performed using the vegan package (Oksanen et al., 2010).

## 3 Results

### 3.1 Field experiments

When cultivated in the absence of pesticides (i.e., Method-1), the leaves were damaged by several noctuid larvae 19 days after the initiation of the field experiments. A few Japanese beetles (*Anomala rufocuprea*, Coleoptera: Scarabaeidae) were also observed on the leaves during the experiments. Ears and tassels were damaged by the Asian corn borer (*Ostrinia furnacalis*, Lepidoptera: Crambidae), corn aphids (*Rhopalosiphum maidis*, Hemiptera: Aphididae), and bird cherry-oat aphids (*R. padi*, Hemiptera: Aphididae).

**Figure 2:**
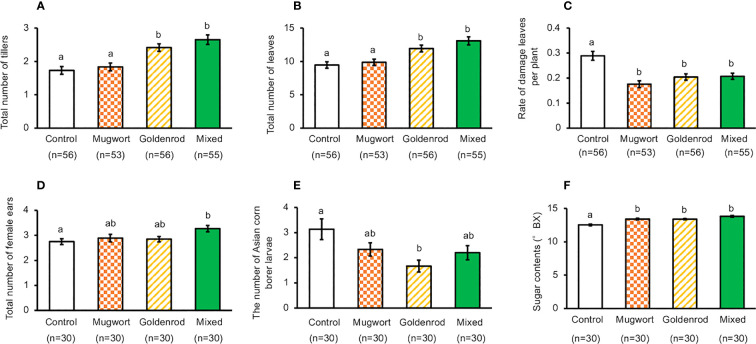
Performance of exposed and control maize plants cultivated using Method-1 under field conditions. **(A)** Number of tillers, **(B)** Number of leaves, **(C)** Rate of damaged leaves, **(D)** Total number of ears, **(E)** Number of Asian corn borer larvae, and **(F)** Seed sugar content. Data are means ± standard errors. Different letters indicate significant differences among treatments (Tukey’s Honestly Significant Difference test, α = 0.05).

Under cultivation by Method-1, the total number of tillers and leaves increased significantly in maize plants treated with goldenrod volatiles or with the volatile mixture, compared to the control plants. However, these numbers were not significantly different between maize plants treated with mugwort volatiles and the controls (ANOVA, for total number of tillers, *F*_3,212_ = 13.11, *P* < 0.001, for total number of leaves, *F*_3,212_ = 10.99, *P* < 0.001) (**Figures 2A, B**).

In addition, the percentage of damaged leaves decreased significantly in plants exposed to any of the three volatile sources, when compared with control plants (ANOVA, *F*_3,212_ = 12.38, *P* < 0.001, **Figure 2C**). Compared with the control plants, the number of ears increased significantly only in plants treated with the mixture of volatiles (ANOVA, *F*_3,212_ = 3.11, *P* = 0.0273, **Figure 2D**). We counted the number of Asian corn borer larvae and found that their number in the ears of the plants exposed to goldenrod volatiles was significantly lower than that in control plants, but such a decrease was not detected in the ears from other treated plants (ANOVA, *F*_3,116_ = 3.927, *P* = 0.0104, **Figure 2E**).

Number of kernels, ear length, and kernel weight of the top ears collected from plants cultivated using Method-1 were not significantly different between treated and control plants (**Supplementary Figure S1**) (ANOVA, for total number of seeds, *F*_3,116_ = 0.757, *P* = 0.520, for ear length, *F*_3,116_ = 0.348, *P* = 0.791, for dry weight of seeds, *F*_3,116_ = 3.608, *P* = 0.155).

**Figure 3:**
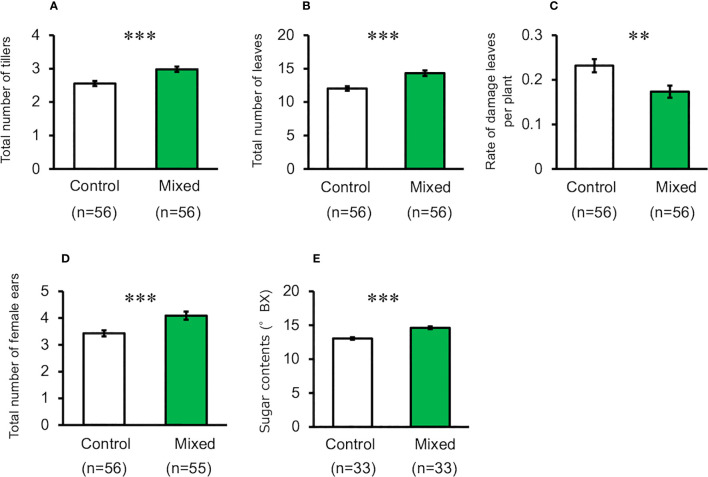
Performance of exposed and control maize plants cultivated using Method-2 under field conditions. **(A)** Number of tillers, **(B)** Number of leaves, **(C)** Rate of damaged leaves, **(D)** Total number of ears, and **(E)** Seed sugar content. Data are means ± standard error. ** indicates 0.01 > *P* > 0.001, *** indicates 0.001 > *P*, as per the *t*-test.

When maize plants exposed to the mixture of volatiles were cultivated using pesticides and by picking multiple ears (i.e., Method-2), the number of tillers, leaves, and ears of treated plants were significantly higher than those of the control plants (*t*-test, for total number of tillers, *d.f.* = 110, *t* = 3.920, *P* < 0.001, for total number of leaves, *d.f.* = 110, *t* = 4.228, *P* < 0.001, for total number of ears, *d.f.* = 109, *t* = 3.534, *P* < 0.001, **Figures 3A, B, D**). The levels of damage in volatile-treated plants were significantly lower than those in the control plants (*t*-test, *d.f.* = 110, *t* = 2.903, *P* = 0.004, **Figure 3C**). Kernel number and dry weight, and top-ear length of treated plants were not significantly different from those of the control plants (**Supplementary Figure S2**) (*t*-test; total number of seeds, *d.f.* = 102, *t* = 0.682, *P* = 0.497; dry weight of seeds, *d.f.* = 64, *t* = 0.406, *P* = 0.686; ear length *d.f.* = 102, *t* = 1.609, *P* = 0.111).

### 3.2 Sugar content and taste assessment

Seedling exposure to mugwort or goldenrod volatiles, or to the mixture of volatiles resulted in a significant increase of seed sugar content in the top ears, compared with control plants when cultivated using Method-1 (ANOVA, *F*_3,116_ = 20.88, *P* < 0.001, **Figure 2F**). Similarly, when cultivated using Method-2, seed sugar content in the top ears of treated plants was also significantly higher than that in the control plants (*t*-test, *d.f.* = 64, *t* = 6.204, *P* < 0.001, **Figure 3E**).The results of the tasting experiments revealed that 16 persons believed maize seeds from plants exposed to the mixture of volatiles to be sweeter than those from the control plants, while five persons believed that seeds from the control plants were sweeter than those from the treated plants (binomial test, *P* = 0.027).

### 3.3 Feeding experiment

**Figure 4:**
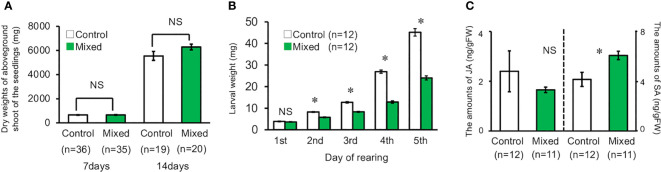
**(A)** Dry weight of the aboveground shoot of seedling reared under laboratory conditions on the 7^th^ and 14^th^ day. **(B)** Weight of second instar *M. separata* larvae reared on plants under laboratory conditions for five days. **(C)** Amount of JA and SA in exposed and control plants grown under laboratory conditions. Data are means ± standard error. * indicates 0.05 > *P* as per the *t*-test. NS means not significant (*P* > 0.05).

The dry weight of the aboveground shoot of volatile-treated seedlings was not significantly different from that of the control seedlings (*t*-test, for seven days, *d.f.* = 69, *t* = 0.091, *P* = 0.93, for 14 days, *d.f*. = 37, *t* = 1.685, *P* = 0.100, **Figure 4A**). In contrast, the weight of *M. separata* larvae that fed on the seedlings exposed to the volatiles mixture was significantly lower than that of the larvae feeding on the control seedlings, except for day one of the experimental period (*t*-test, for day one, *d.f.* = 22, *t* = 0.901, *P* = 0.378; for day two, *d.f.* = 22, *t* = 8.380, *P* < 0.001; for day three, *d.f.* = 22, *t* = 10.485, *P* < 0.001; for day four, *d.f.* = 22, *t* = 14.612, *P* < 0.001; for day five, *d.f.* = 22, *t* = 10.688, *P* < 0.001, **Figure 4B**).

### 3.4 Quantification of JA and SA

The mean JA content in the seedlings exposed to the volatile mixture was not significantly higher than that in the control seedlings (*t*-test, *d.f.* = 21, *t* = 0.761, *P* = 0.455, **Figure 4C**). In contrast, the mean SA content in the volatile-treated seedlings was significantly higher than that in the control seedlings (*t*-test, *d.f.* = 21, *t* = 2.1943, *P* = 0.0396, **Figure 4C**).

### 3.5 Volatiles

Eighteen compounds were detected in the headspace of cut mugwort plants, on Day-1 and Day-2. The major compounds on Day-1 were β-pinene, β-myrcene, (*Z*)-3-hexenyl acetate, caryophyllene, (*E*)-β-farnesene, and humulene, while those on Day-2 were *β*-pinene, caryophyllene, and (*E*)-*β*-farnesene (**Table 1**). The total amount of volatiles detected on Day-2 was smaller by ca.15% than that detected on Day-1 (*t*-test, *P* = 0.063).

**Table 1:** Volatile organic compounds in the headspace of artificially damaged mugworts, goldenrods and a mixture of two species.

Compound	Peak area/gFW (×10^5^)					
	Mugwort		Goldenrod		Mixed	
	Day1	Day2	Day1	Day2	Day1	Day2
(*Z*)-3-Hexenol	17.7 ± 8.4	0	0	0	0	0
*α*-Pinene	26.6 ± 19.1	13.0 ± 11.5	52.4 ± 30.3	61.1 ± 30.5	236.6 ± 184.8	55.1 ± 21.1
*β*-Pinene	70.4 ± 46.8	25.1 ± 21.5	254.0 ± 146.7	16.2 ± 8.1	108.5 ± 64.8	39.8 ± 20.0
*β*-Myrcene	61.9 ± 22.0	11.5 ± 9.4	1026.3 ± 592.6	26.0 ± 13.0	543.4 ± 174.8	26.3 ± 9.0
(*Z*)-3-Hexenyl acetate	148.3 ± 48.6	0	61.1 ± 35.3	0	39.0 ± 20.1	0
Limonene	22.9 ± 7.0	0	917.1 ± 529.5	66.4 ± 33.2	537.1 ± 186.2	39.4 ± 16.4
Eucalyptol	23.3 ± 3.8	0	0	0	0	0
(*E*)-*β*-Ocimene	12.3 ± 5.7	0	139.2 ± 80.4	0	56.1 ± 23.3	0
1,4-Hexadiene, 5-methyl-3-(1-methylethylidene)	9.2 ± 3.9	0	0	0	12.1 ± 6.8	0
Borneol	16.3 ± 5.8	0	24.3 ± 14.0	0	35.8 ± 14.7	0
Bornyl acetate	0	0	482.7 ± 278.7	0	359.6 ± 153.8	0
*γ*-Elemene	0	0	0	4.8 ± 2.4	0	0
Terpinolene	0	0	26.1 ± 150.9	0	61.8 ± 16.2	0
*β*-Ylangene	0	0	0	63.5 ± 31.8	0	0
*β*-Caryophyllene	407.1 ± 191.9	54.4 ± 28.5	273.9 ± 158.2	0	312.1 ± 182.8	41.5 ± 13.0
Aromandendrene	8.4 ± 2.8	0	0	0	0	0
*β*-Copaene	0	0	133.8 ± 77.3	20.2 ± 10.1	208.2 ± 102.1	8.1 ± 1.6
(*E*)-*β*-Farnesene	273.3 ± 122.2	41.1 ± 13.8	0	0	126.1 ± 31.2	9.8 ± 4.1
Humulene	96.9 ± 33.4	17.5 ± 8.4	0	0	tr	16.8 ± 6.0
Germacrene-D	31.5 ± 13.6	1.8 ± 6.8	65.1 ± 37.6	147.5 ± 73.7	158.9 ± 53.0	39.9 ± 26.9
Zingiberene	39.4 ± 13.6	3.5 ± 4.5	0	0	tr	0
*α*-Farnesene	16.6 ± 7.8	15.4 ± 1.2	0	0	0	0
*γ*-Muurolene	7.8 ± 4.5	8.2 ± 0	99.7 ± 57.5	11.6 ± 5.8	0	6.6 ± 2.0

Date are represented by mean ± standard error. Tr means a trace amount.

In the headspace of cut goldenrod plants, 13 and 9 compounds were detected on Day-1 and Day-2, respectively. The major compounds on Day-1 were β-pinene, β-myrcene, limonene, bornyl acetate, and caryophyllene, while those on Day-2 were α-pinene, β-pinene, β-myrcene, limonene, β-ylangene, β-copaene, and germacrene-D (**Table 1**). The total amount of volatiles detected on Day-2 was significantly lesser than that on Day-1 (ca.12%) (*t*-test; *P* = 0.019).

Meanwhile, 14 and 10 compounds were detected in the headspace of the volatile mixture of the two cut weeds used herein, on Day-1 and Day-2, respectively. The major compounds on Day-1 were α-pinene, β-myrcene, limonene, bornyl acetate, caryophyllene, β-copaene, and germacrene-D. Meanwhile, the major compounds detected on Day-2 were α-pinene, β-pinene, limonene, caryophyllene and germacrene-D (**Table 1**). The total amount of volatiles on Day-2 was significantly smaller by ca.11% than that on Day-1 (*t*-test, *P* = 0.012).

**Figure 5:**
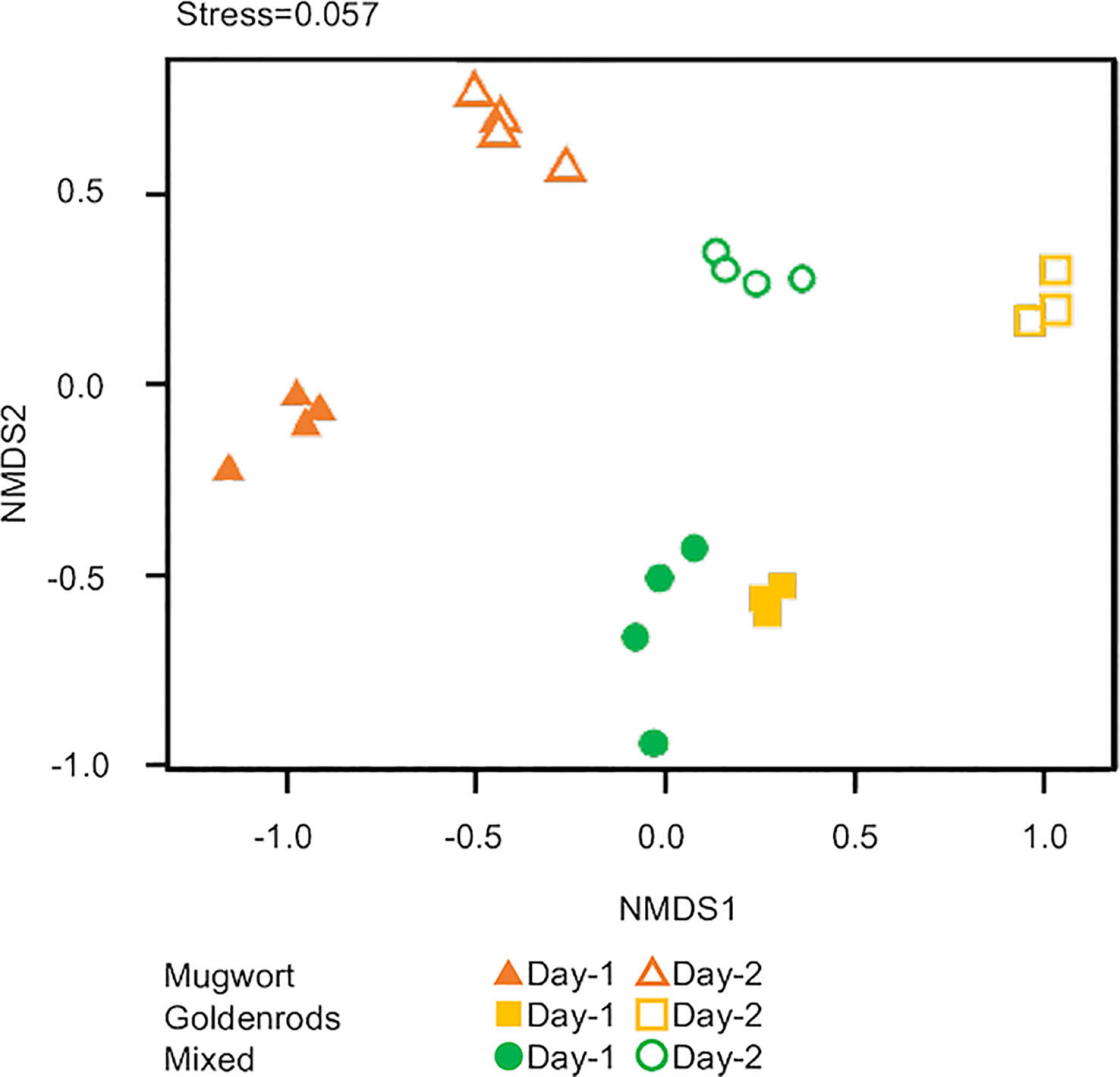
Nonmetric multidimensional scaling (NMDS) ordination of volatiles from three volatile sources.

Lastly, we applied NMDS to all three volatile sources to detect compositional differences. The scent compositions of the three volatile sources used herein differed significantly between volatile sources and among days after treatment (PERMANOVA, volatile source: *d.f.* = 2, pseudo-*F* = 4.52, *P* < 0.01; days: *d.f.* = 1, pseudo-*F* = 9.81, *P* < 0.01; **Figure 5**).

## 4 Discussion

In this study, we evaluated the potential use of weed volatiles in maize cultivation. Our field experiments revealed that exposure to the volatiles emitted by mugworts, goldenrods, and a mixture of the two resulted in the reduction of insect damage to the leaves. Furthermore, under laboratory conditions, a significant reduction in the growth of *M. separata* larvae was observed when maize plants were exposed to the mixture of volatiles, thereby supporting our field results. Overall, we conclude that the leaf defensive responses against herbivores were induced by exposure of weed volatiles. Moreover, the number of Asian corn borer larvae in plant tillers tended to decrease with exposure, indicating that some defensive responses were induced in the tillers as well. It has been reported that soybean plants and their seeds sustain less damage when they are exposed to goldenrod volatiles in the early developmental stage (Shiojiri et al., 2017). Further, the induction of defensive responses in plant-plant communication has been reported in several other plant species under both laboratory and field conditions (for review Karban et al., 2014; Yoneya and Takabayashi, 2014; Meents and Mithöfer, 2020).

The upregulation of resistance in plants is thought to have physiological and ecological costs, and thus trade-offs between upregulating resistance and other traits, such as growth and reproduction, are expected (Karban and Baldwin, 1997; Züst and Agrawal, 2017; Zhang et al., 2020). However, in this study, we did not observe any trade-offs between upregulated resistance and plant growth and/or reproduction as a result of volatile exposure. Rather, in the case of exposure to goldenrod volatiles, in addition to the promotion of resistance, exposure resulted in an increased number of tillers and leaves. Furthermore, the number of ears tended to increase in response to the three volatile sources, although a significant difference relative to controls was detected only when plants were exposed to the mixture of volatiles, suggesting that exposure positively affected reproduction. Shiojiri et al. (2020a) reported that the number of grains increased in a rice plant, in addition to increasing insect resistance upon exposure to weed volatiles. Similarly, sagebrush increased the production of inflorescences and lateral branches when exposed to volatiles emitted from damaged neighboring plants (Karban et al., 2012; Karban, 2017). Furthermore, exposure to volatiles released by *Brassica nigra* on which herbivore eggs were oviposited induced a shift from vegetative growth to flowering in the neighboring conspecific (Pashalidou et al., 2020).

Interestingly, exposure to volatiles increased the amounts of sugars (primary compounds) in maize seeds. As a significantly large number of the participants of tasting experiments reported that seeds from the exposed maize plants were sweeter than those from control plants, volatile exposure might presumably increase the market quality of corn ears. Choh et al. (2006) reported that exposure of lima bean plants to HIPVs from conspecific plants infested with *Tetranychus urticae* increased fructose and glucose in their extrafloral nectary. The biosynthesis of secondary compounds in a plant is affected by the exposure of volatiles in plant-plant communication (Ozawa et al., 2013; Shiojiri et al., 2017; Shiojiri et al., 2020b; Brosset and Blande, 2022). For example, exposure of black and yellow soybean plants to goldenrod volatiles resulted in an increase in seed isoflavones and saponins (Shiojiri et al., 2017; Shiojiri et al., 2020b). Whether the composition of secondary compounds in maize is affected by volatile exposure will be the focus of a future study.

The concentration of SA in maize seedlings increased significantly after exposure to the volatiles mixture but that of JA did not. In tea leaves, SA concentrations were found to increase in response to exposure to indole, a volatile compound (Ye et al., 2020). However, the relationship between increased resistance and SA in maize seedlings in this study remains unclear. Choh et al. (2004) reported that exogenous treatment of lima bean plants with benzo-(1,2,3)-thiadiazole-7-carbothioic acid S-methyl ester (BTH), a functional analog of SA, resulted in a reduction in the number of eggs of two-spotted spider mites (*T. urticae*). In contrast, Zhang et al. (2019) reported that whitefly-HIPVs from tomato plants primed SA-dependent defenses and suppressed JA-dependent defenses to increase the susceptibility of exposed plants to whiteflies. Maize seedlings previously exposed to green leaf volatiles ((*Z*)-3-hexenal, (*Z*)-3-hexenol, and (*Z*)-3-hexenyl acetate) from neighboring plants produced significantly more JA after mechanical damage or subsequent caterpillar regurgitant, but they did not produce more SA in the same experimental design (Engelberth et al., 2004). Thus, the effects of volatile mixtures (defensive and growth performance, sugar content, and increase in SA) on maize seedlings might not be due to the effects of green leaf volatiles.

As expected, volatiles released from mugworts and goldenrods differed in their compositions, and that of their mixture was intermediate. GLVs such as (*Z*)-3-hexenol and (*Z*)-3-hexenyl acetate were found as minor compounds in mugwort volatiles, whereas only (*Z*)-3-hexenyl acetate was found in goldenrod volatiles as a minor compound. In contrast, bornyl acetate, terpinolene, and *β*-copaene were found only in goldenrod volatiles of Day-1. Some of these volatiles would be involved in the induction of specific responses by goldenrod volatiles (e.g., increase in leaf number) in maize seedlings. Further studies are needed to determine the effects of each compound on seedlings.

It is important to note that the number of ears increased significantly in both cultivation methods. While this indicates fitness in the case of Method-1, this finding implies the possibility of the increasing of commercial production in the case of Method 2. Our results suggest that even when maize plants are commercially grown using the conventional method, exposure of seedlings to the mixture of volatiles or goldenrod volatiles can increase the performance (such as the number of leaves, tillers, ears, and seed sugar content) and herbivore resistance of maize plants.

## Data availability statement

The original contributions presented in the study are included in the article/**Supplementary Material**. Further inquiries can be directed to the corresponding author.

## Author contributions

YS, SIs, and KS designed and conducted the experiments. SIz and TY conducted the experiments. YS, SIs, KS, and JT analyzed the data and wrote the manuscript. All authors contributed to the article and approved the submitted version.

## Supplementary material

Supplementary Figure 1 Performance of exposed and control maize plants cultivated using Method-1 under field conditions. **(A)** Number of seeds of the top ear, **(B)** Length of top ears, **(C)** Seed dry weight, and **(D)** Relative rate of mature seeds. Data are means ± standard error. NS means not significant (P > 0.05).

Supplementary Figure 2 Performance of exposed and control maize plants cultivated using Method-2 under field conditions. **(A)** Number of seeds of the top ear, **(B)** Length of top ears, and **(C)** Seed dry weight. Data are means ± standard error. NS means not significant (P > 0.05).
