# Supplementary figures and images for: The exposure of field-grown maize seedlings to weed volatiles affects their growth and seed quality
Source: Front Plant Sci. 2023 Aug 15;14:1141338. doi: 10.3389/fpls.2023.1141338 (PMC10464949; doi:10.3389/fpls.2023.1141338)

(A)

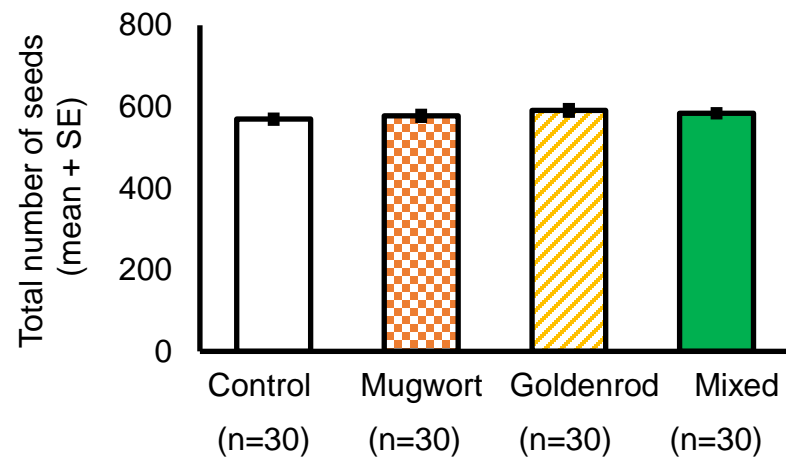

(B)

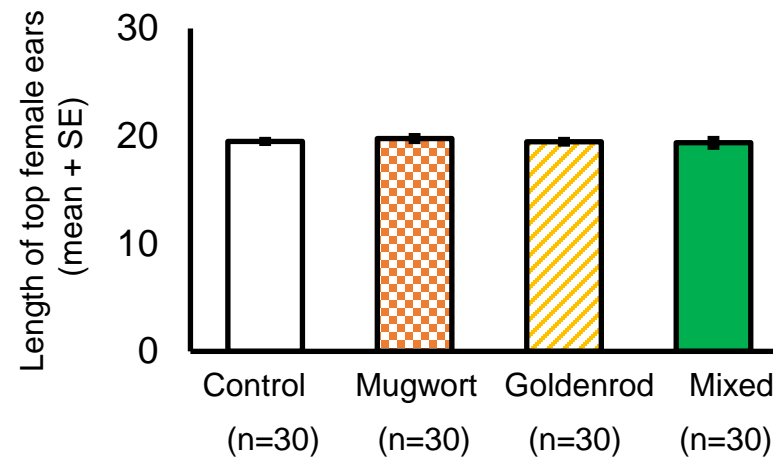

(C)

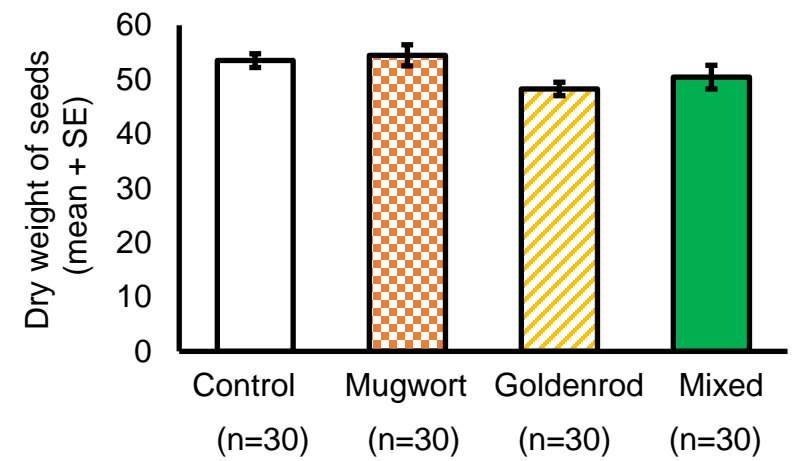

(D)

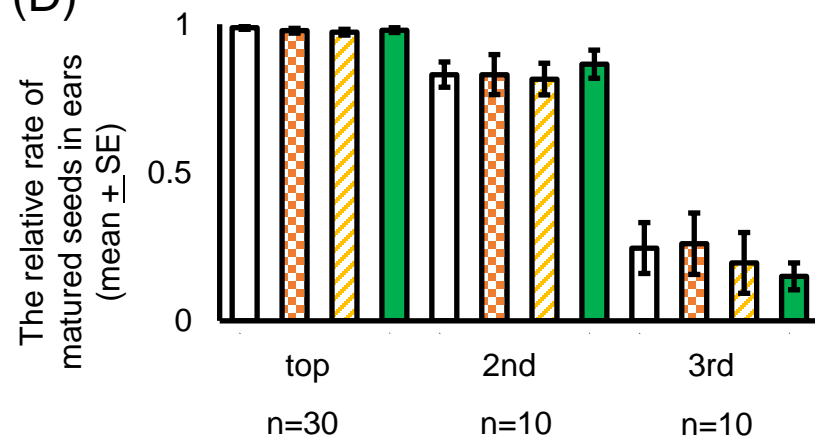

Figure S1

(A)

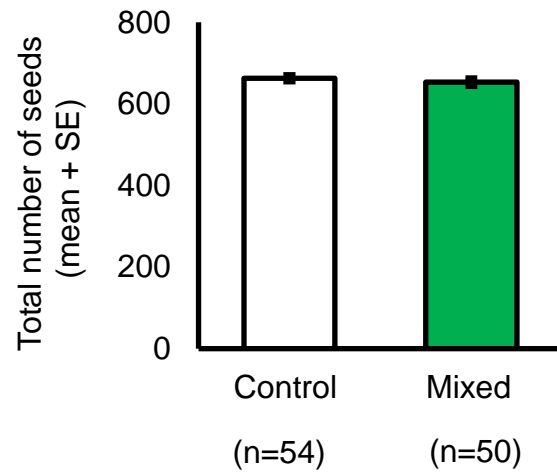

(B)

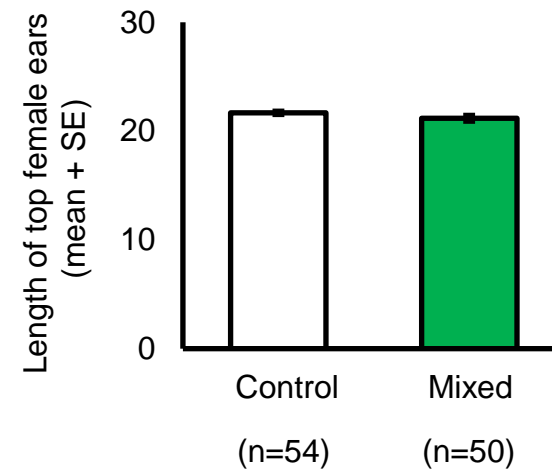

(C)

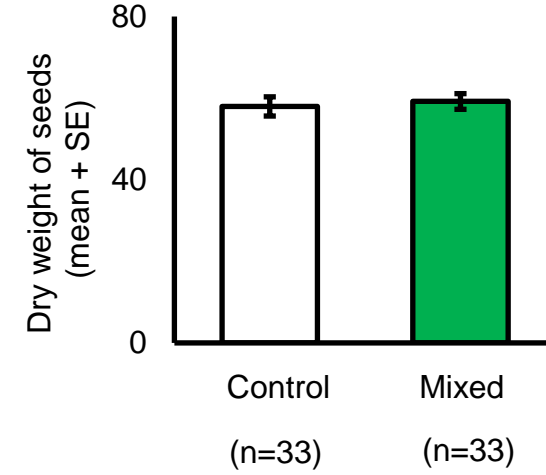

Figure S2

Supplement: Supplementary Figure 1 — Performance of exposed and control maize plants cultivated using Method-1 under field conditions. (A) Number of seeds of the top ear, (B) Length of top ears, (C) Seed dry weight, and (D) Relative rate of mature seeds. Data are means ± standard error. NS means not significant (P > 0.05). [file DataSheet_1.pdf]
